# Identification of selective sweep and associated QTL traits in Iranian *Ovis aries* and *Ovis orientalis* populations

**DOI:** 10.3389/fgene.2024.1414717

**Published:** 2024-12-19

**Authors:** Sadegh Taheri, Ali Javadmanesh, Saeed Zerehdaran

**Affiliations:** Department of Animal Science, Faculty of Agriculture, Ferdowsi University of Mashhad, Mashhad, Iran

**Keywords:** domestic and wild sheep, iHS, RSB, selective sweep, QTL, XP-EHH

## Abstract

**Introduction:**

Identifying genomic regions under selection is the most challenging issue for improving important traits in animals. Few studies have focused on identifying genomic regions under selection in sheep. The aim of this study was to identify selective sweeps and to explore the relationship between these and quantitative trait loci (QTL) in both domestic and wild sheep species using single nucleotide polymorphism markers (SNPs).

**Methods:**

Genomic data were obtained from the NextGen project, which included genotyping 20 domestic and 14 wild sheep using the Illumina Ovine SNP50K BeadChip. The XP-EHH, iHS, and RSB methods were employed to detect signatures of positive selection.

**Results:**

The results of the iHS method indicated 405 and 275 selective sweeps in domestic and wild sheep, respectively. Additionally, RSB and XP-EHH analyses revealed approximately 398 and 479 selective sweeps in domestic and wild sheep, respectively. Some of the genes associated with important QTL traits in domestic sheep include *ADGRB3, CADM1, CAPN2, GALNT10, MTR, RELN*, and *USP25*, while in wild sheep, the relevant genes include *ACAN, ACO1, GADL1, MGST3*, and *PRDM16*. Selective sweeps identified in domestic sheep were associated with body weight, muscle weight, milk protein percentage, and milk yield. In contrast, selective sweeps found in wild sheep were linked to average daily gain, bone weight, carcass fat percentage, and dressing percentage.

**Discussion:**

These results indicate that selection by humans and the environment have largely progressed in harmony, highlighting the importance of both economic and environmental traits for survival. Additionally, the identification of potential candidate genes associated with economic traits and genomic regions that have experienced selection can be utilized in sheep breeding programs. However, due to the incomplete information regarding the functional annotation of genes in sheep and the limited sample size, further research with a larger sample group is essential to gain a deeper understanding of the candidate genes linked to economic traits in both domestic and wild sheep. Advancing knowledge in this area can significantly enhance the effectiveness of breeding strategies. The quantitative trait loci identified in this study have the potential to be incorporated into breeding plans for both domestic and wild sheep.

## 1 Introduction

Animal domestication has significantly influenced human life. Due to their high adaptability to various climates and diets, sheep were among the first animals to be domesticated (Zeder, 2008). Evidence suggests that Iran was a world center for farm animal domestication (Zeder, 1999). Archaeological findings indicate that sheep were domesticated approximately 9,000 years ago in what is now northern Iraq and parts of Iran (Kijas et al., 2009; Zeder, 1999). The wild sheep of Iran include three species: Vignei sheep (*Ovis vignei*), Asian mouflon (*O. orientalis*), and hybrids with varying chromosome numbers, which are found in the eastern and western regions and the Alborz Mountain range of northern Iran. Iranian wild sheep are recognized as the ancestors of domestic sheep worldwide, which may account for the diversity of domestic sheep breeds in Iran (Hamadalahmad et al., 2020). Domestic sheep are vital to Iran’s agricultural economy and the livelihoods of its people, serving as a source of milk, meat, and other byproducts. To effectively manage the genetic resources of both wild and domestic animals, it is essential to understand the characteristics of different breeds. These include population size and structure, geographical distribution, optimal environmental conditions for production and performance, and levels of genetic diversity within and among breeds (Javadmanesh et al., 2022). Domestication has significantly altered the behavioral and morphological traits of these animals. In addition to domestication, selective breeding for increased production or specific behavioral and morphological traits has led to the emergence of highly divergent modern breeds (Flori et al., 2009). Post-domestication selection for economic and morphological traits can have lasting effects on the genomes of sheep. This selection, combined with natural adaptation to local environments, has resulted in the development of over a thousand distinct breeds of sheep (Kijas et al., 2012). These genetic features, along with the extensive genomic information related to economic traits, offer an opportunity to identify loci that are under selection (Hayes et al., 2008). Selection can lead to specific changes in both selected and neutral sites, with selection signatures—genomic footprints left by selection—used to identify these target areas (Kreitman, 2000). The recent availability of genomic data from domestic animals, coupled with advances in statistical tools, has made it possible to detect these signatures in specific species (Dong et al., 2013). Identifying selection signatures is a key focus for evolutionary geneticists, as it provides insight into the evolutionary processes that shape genomes and translates genomic data into functional information about important regions (Schlötterer, 2003). Several statistical methods are available for the investigation of selection signatures, including the integrated haplotype homozygosity score (iHS) (Voight et al., 2006), cross-population extended haplotype homozygosity (XP_EHH) (Sabeti et al., 2007), and the ratio of extended haplotype homozygosity between populations (RSB) (Tang et al., 2007). Advances in livestock genomics research have led to the recent development of QTL databases linked to the latest reference genomes of various livestock species, such as sheep, cows, pigs, and fish (Hui et al., 2021). These databases provide precise locations of genes and genomic regions associated with known traits in livestock species. As a result, researchers can use these fine-mapped QTL traits to identify selection signatures related to desired traits in livestock species, which could be useful for breeding programs (Zeraatpisheh et al., 2023).

The current study aimed to detect the regions under natural and artificial selection in two wild and domestic Iranian sheep species using iHS, XP-EHH, and RSB methods. The communication between selective sweep and QTL traits was also identified using single nucleotide markers (SNP).

## 2 Materials and methods

### 2.1 Sampling and genotyping

The data utilized in this research were retrieved from the NextGen project (http://projects.ensembl.org/nextgen/). The objective of this project is to assess the genetic diversity of some domestic and wild species such as sheep, goat, and cattle by utilizing whole-genome sequencing and genotyping by microarray. For this particular study, samples were collected from 20 Iranian domestic (*O. aries*) and 14 Iranian wild sheep (*O. orientalis*). Samples were taken according to the Helsinki Declaration of 1975 (as revised in 2008) concerning animal rights, and this study was approved by the Animal Ethical Committee (3/51431), Ferdowsi University of Mashad, Mashhad, Iran (Taheri et al., 2022). Ear tissue samples were used for DNA extraction using a Macherey Nagel NucleoSpin 96 Tissue kit, following the manufacturer’s instructions, as per Alberto et al. (2018). To integrate the whole-genome sequencing data with the variations in each animal’s entire genome, the NextGen project’s equivalent of the SNP50 BeadChip array was obtained from every individual utilizing PLINK v1.9 software.

### 2.2 Quality check and PCA analyses

To ensure the quality of the data, the PLINK version 1.9 application (Purcell et al., 2007) was used to perform quality control for the two populations. Individuals with a genotyping call rate of less than 99% were removed. Additionally, SNPs with a genotyping call rate of less than 99%, a minor allele frequency (MAF) of less than 5%, and a significant deviation from Hardy–Weinberg equilibrium (HWE ) (*p* < 10^6^) were excluded (Taheri et al., 2023). Following this filtering process, the genotype data for both populations were combined, and common SNPs between them were selected.

To investigate the structures of the domestic and wild sheep populations and to identify animals that did not fit into their respective populations, principal component analysis (PCA ) was conducted using a relative genomic matrix. PLINK version 1.9 was employed to perform this analysis.

### 2.3 Detection of selection sweep and significant SNPs

The three methods used in this study—iHS, RSB, and XP-EHH—are basically the same, based on the concept of extended haplotype homozygosity (EHH), to increase the accuracy in identifying selection signatures. iHS is designed for use on a specific population, while XP-EHH and RSB are for comparing differences in selection between two populations. Three statistics were calculated using the EHH concept developed by Sabeti et al. (2002).

iHS analysis is a statistical method for identifying haplotype-based selection sweeps which involves the calculation of EHH from both ancestral (iHHA) and derived (iHHD) biallelic SNPs. iHS is a within-population value based on the rate of LD decay at a certain variant and is calculated as per Voight et al. (2006):

$$iHS=\ln\;\left(\frac{iHHA}{iHHD}\right).$$

The “rehh” package (Gautier and Vitalis, 2012) in R was utilized to separately estimate iHS values for SNPs with MAF ≥0.05 within domestic and wild populations. Large positive or negative values of iHS show long haplotypes harboring ancestral or derived alleles, respectively.

RSB is also calculated based on EHH. However, unlike iHS, RSB involves comparing the EHH patterns of the common alleles (as “iES”) between the two populations. Tang et al. (2007) defined RSB as:

$$\ln\;\left(RSB\right)=\ln\left(\frac{iESpop1}{iESPOP2}\right).$$

To estimate RSB values, various iES statistics were estimated using the “rehh” package during the iHS analyses in each of the domestic and wild populations, as described previously. The RSB values were normalized in the R environment. Since the RSB value is directional, positive values indicate selection in the wild population, while negative values indicate selection in the domestic population.

XP-EHH was also utilized to identify any recent selection sweeps within the sheep genome. This method is based on evaluating LD decay across the genome and EHH values. For biallelic SNPs with alleles A and a, EHH was calculated using the following formula (Sabeti et al., 2002):

$$EHH=\frac{\sum_{i=1}^{h_{x}}hx\left(ni\;2\;\right)}{\left(na\;2\;\right)\;\left(nA\;2\;\right)},$$

where *nA* and *na* represent haplotype counts with alleles *A* and *a*, respectively, *ni* is the count for the *i* ^th^ haplotype within a sub-population, and *hx* represents the number of distinct haplotypes in a genomic area up to a distance *x* from the core locus. All SNPs located within one Mbp interval up- and downstream of a given SNP were taken into account, and EHH was integrated within these intervals for two groups. XP-EHH was estimated according to Sabeti et al. (2007). The highest negative and positive values indicate locations under selection in domestic and wild sheep populations, respectively. The “rehh” software was utilized to estimate XP-EHH, and the top 5% of estimated values were selected for further analysis using both domestic and wild populations (Tao et al., 2020). To increase the power of detecting selection signatures, we selected the overlapping areas between RSB, XP-EHH, and iHS techniques in domestic and wild sheep.

After identifying selection signatures using the three methods, similar locations in both populations were identified separately. The candidate genes related to these common locations were then selected using PLINK V1.9 (Purcell et al., 2007), utilizing a gene list provided from the sheep reference genome (http://ncbi.nlm.nih.gov/genome/?term=sheep).

### 2.4 Gene ontology and KEGG pathway analyses of genes related to significant SNPs

To identify gene ontology terms and significant metabolic KEGG pathways associated with genes related to significant SNPs, ClueGo version 2.5.6 was used, which is a Cytoscape plug-in that provides biological interpretations of genes (Bindea et al., 2009). Significant genes were then subjected to functional enrichment analysis using the Cytoscape application (Saito et al., 2012) and ClueGO 2.5.6 plug-in (Bindea et al., 2009). Official Gene Symbol was used as the input parameter, and *O. aries* was selected as the background organism. After Bonferroni correction for multiple testing, *p*-values <0.05 were considered statistically significant.

### 2.5 Extraction of QTL traits associated with genes related to significant QTLs

Hu et al. (2022) utilized the AnimalQTL database (www.animalgenome.org/cgi-bin/QTLdb/OA/index) to locate sheep QTL traits previously reported. They then compared the genes identified in regions of high three methods with these QTL traits to determine whether any traits were under selection in Iranian domestic and wild sheep.

## 3 Results

### 3.1 Quality control and PCA analyses

**FIGURE 1:**
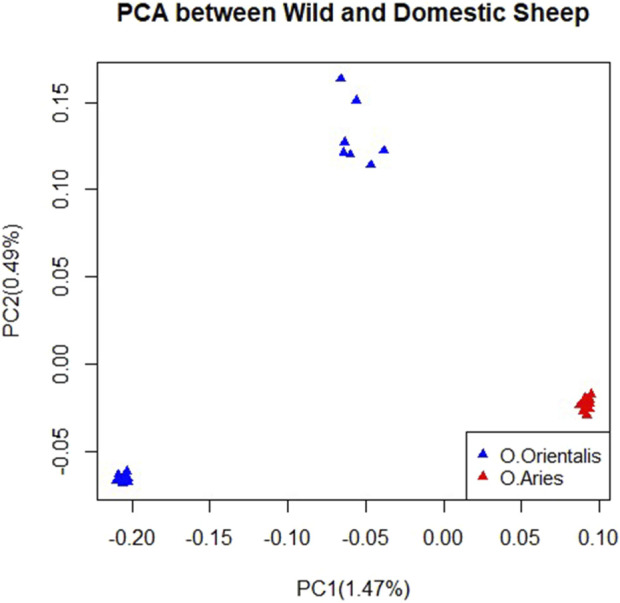
Clustering based on principal component analysis (PCA) using SNP data for domestic and wild sheep.

After performing quality control on the genomic data, we selected 33,167 and 35,479 SNPs for further analysis in domestic and wild sheep, respectively. To analyze the genetic connection between domestic and wild sheep and characterize their divergence, we conducted principal component analysis (PCA). The results of the PCA showed that domestic and wild sheep were clearly divided as shown in the first and second principal component factors. Furthermore, the results showed that the samples of domestic sheep had lower within-population variation, while the wild sheep had more within-population variation, indicating two possible subpopulations (Figure 1).

### 3.2 Detection of selection sweep, significant SNPs, and their related genes

**FIGURE 2:**
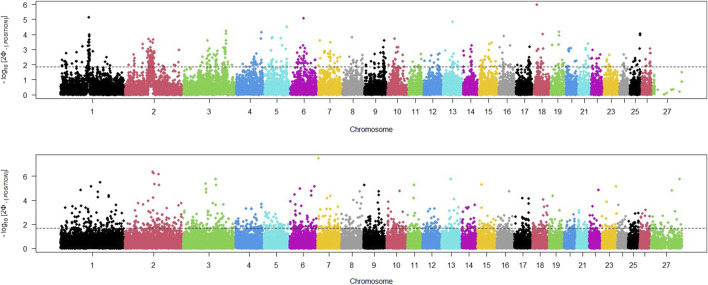
Distribution of iHS values across the genome in wild (above) and domestic (below) sheep; threshold indicates the top 5% SNPs with the largest iHs for each population.

The study utilized three different statistical tests (iHS, RSB, XP_EHH) to detect any regions of the genome that had potentially undergone recent selection. The iHS test was applied to SNPs across the genome of both domestic and wild sheep populations, and a Manhattan plot was generated to display the distribution of iHS signals across all chromosomes (Figure 2). We adjusted the threshold value to identify the top 5*%* of SNPs with the highest iHs for each population. The Manhattan plot for the wild sheep population indicated 275 locations with the highest iHS values (iHS>1.91) on chromosomes 13, 18, 6, and 1. The Manhattan plot for the domestic sheep population revealed 405 regions with the highest signals (iHS>1.86) on chromosomes 7, 2, and 3.

**FIGURE 3:**
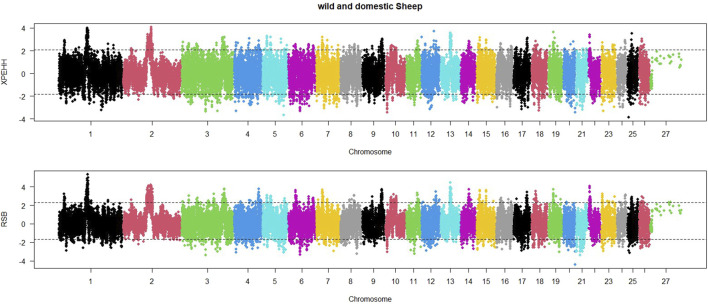
Distribution of XP-EHH and RSB across the genome between domestic (lower than 0) and wild (higher than 0) sheep. Thresholds indicate the top 5% SNPs for each population.

In order to identify selective sweep in the wild and domestic populations, an EHH-based RSB method was also utilized. The distribution of RSB values across sheep autosomal chromosomes is shown in Figure 3. Based on regions with RSB values lower than −1.92, 421 regions were found to be under selection in the domestic sheep population. The lowest RSB values were observed on chromosomes 20, 11, and 6. Similarly, significant positive RSB values (RSB>2.05) were identified in 376 regions in the wild sheep population. The highest RSB values were found on chromosomes 1, 2, and 13.

Additionally, a Manhattan plot for XP-EHH values within the sheep genome was generated (Figure 3). Based on regions with XP-EHH values lower than −2.04, 467 regions were identified as under selection in the domestic sheep population. The most negative XP-EHH values were observed on chromosomes 25, 5, and 10. Similarly, according to significant selection signatures for XP-EHH values higher than 1.97, 490 regions were detected in the wild sheep population. The highest XP-EHH values were found on chromosomes 1, 2, and 12.

**FIGURE 4:**
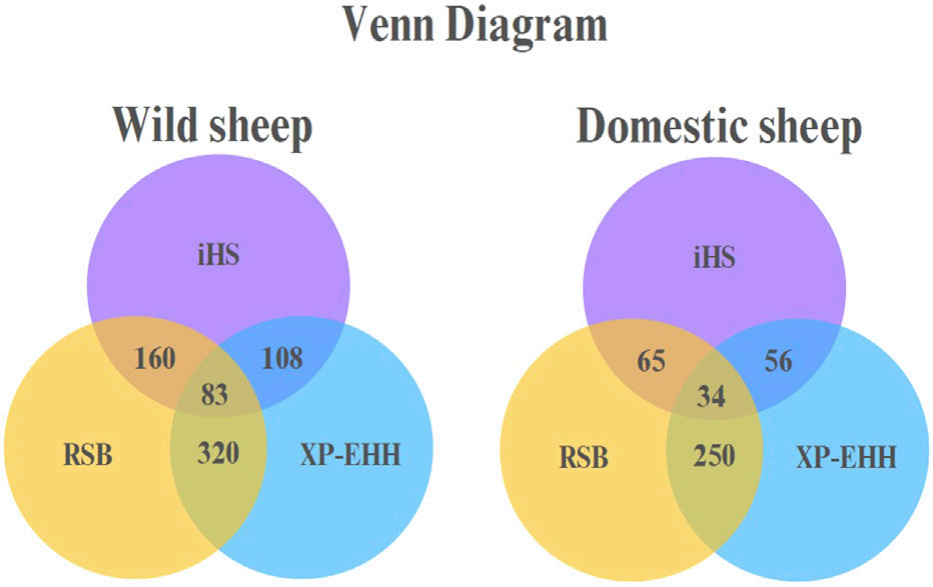
Venn diagram in domestic and wild sheep populations representing overlapping regions between iHS, RSB, and XP-EHH.

A Venn diagram for the domestic and wild sheep populations indicated that 34 genes in the domestic sheep and 83 genes in the wild sheep were shared by all three methods (Figure 4).

### 3.3 Gene ontology and KEGG pathway analyses of genes related to significant SNPs and associated QTL traits

We discovered that overlapping genes between the iHS, RSB, and XP-EHH methods were related to various processes in domestic (cell adhesion molecules, calcium-dependent cysteine-type endopeptidase activity, and cytoplasm) and wild (metabolic pathways, nucleotide metabolism, and glutamatergic synapse) sheep (Table 1).

**TABLE 1:** GO terms and pathways along with the *p*-value of genes related to significant SNPs in domestic and wild sheep populations.

Category	Term	*p*-value	Gene
Wild sheep			
CC	Cytosol	0.006	*ZNF395, RALGAPA2, COPB1, RAP1GDS1, UAP1, ELP3, C9ORF72, PRDM16, UBR5, SPAG1, NEK1, TAF6, ACO1, RGS22, DCAF13, SH3GL1, and PLIN5*
BP	Positive regulation of GTPase activity	0.007	*RALGAPA2, RAP1GDS1, NF1, and C9ORF72*
CC	Presynapse	0.024	*KCNJ10, C9ORF72, and SH3GL1*
KEGG	Nucleotide metabolism	0.028	*UCK2, ENTPD6, and AK8*
CC	Glutamatergic synapse	0.037	*NAPB, ACAN, RNF19A, and SH3GL1*
BP	Regulation of phosphoprotein phosphatase activity	0.046	*TIPRL and TSC1*
KEGG	Metabolic pathways	0.050	*UCK2, PAH, MGST3, PRDM16, ENTPD6, GADL1, ACO1, UAP1, GALNTL6, and AK8*
Domestic sheep			
MF	Calcium-dependent cysteine-type endopeptidase activity	0.004	*CAPN13, CAPN8, and CAPN2*
CC	Cytoplasm	0.005	*TENM4, KCNIP1, RALGAPA1, SPATS2, RANBP17, MTR, CHD3, ARHGAP44, LOC101121185, RELN, XPOT, ELMO1, ARHGEF3, CAPN2, BBOF1, CPNE2, SDCCAG8, EIF4G1, and TRIM67*
CC	Golgi membrane	0.022	*GALNT14, LOC101112819, RTN1, COPG2, and GALNT10*
BP	Activation of cysteine-type endopeptidase activity involved in apoptotic process	0.023	*DLC1, HIP1R, and PML*
KEGG	Other types of O-glycan biosynthesis	0.024	*GALNT14, GXYLT1, and GALNT10*
BP	O-glycan processing	0.036	*GXYLT1 and GALNT10*
MF	SH3 domain binding	0.038	*ELMO1, HIP1R, and HCLS1*
BP	Negative regulation of protein ubiquitination involved in ubiquitin-dependent protein catabolic process	0.042	*HFE and PML*
BP	Negative regulation of angiogenesis	0.046	*ADGRB3, PTPRM, and PML*
MF	GTPase activator activity	0.048	*ARHGAP44, LOC101109993, RALGAPA1, and DLC1*
KEGG	Cell adhesion molecules	0.049	*CADM1, NEGR1, PTPRM, and NRCAM*
MF	SUMO binding	0.050	*USP25 and PML*

CC, cellular component, BP, biological process, MF, molecular function.

The quantitative trait locus report indicated that significant genes identified in the domestic sheep were connected to crucial traits such as body weight, bone density, bone weight, carcass fat percentage, and muscle weight. Additionally, candidate genes identified in the wild sheep were linked to average daily gain, milk fat percentage, milk yield, and muscle weight (Table 2) (more details in Supplementary Tables 1, 2).

**TABLE 2:** QTL traits associated with genes related to significant SNPs along with their genomic locations in domestic and wild populations.

CHR	Gene	SNP	SNP position	QTL
Domestic sheep				
1	NEGR1	ss1113654939	50204860	BONE_WT, BONEP, FA-C20:5, FA-C22:5, FATP, LMYP, MUSWT, and PUFA
1	USP25	ss1116150477 ss1116154441	152500264 152601830	BONE_WT, BONEP, FA-C20:5, FA-C22:5, FATP, FLYD, LMYP, MUSWT, and PUFA
4	COPG2	ss1129997095 ss1129997769	103086813 103151963	CVFD_PRI
4	NRCAM	ss1129534548	55685078	CVFD_PRI
4	RELN	ss1129484817 ss1129485872	50358993 50458109	CVFD_PRI
5	GALNT10	ss1130834348	68288153	FA-C16:1
7	RTN1	ss1133283846	74925794	CVFD_PRI, SL
9	ADGRB3	ss1134565186 ss1134567992	5336181 5535614	LMA and MUSWT
12	CAPN2	ss1137368707	29531925	BDENS, FATP, LMYP, and MY
12	SDCCAG8	ss1137447517	36847011	BDENS, FATP, LMYP, and MY
15	CADM1	ss1139557872	28497013	FECGEN
17	HIP1R	ss1141565009	61655059	BDENS and SCS
18	PML	ss1141994265	32479250	FA-C20:1, FATP, SL, and TESTWT
23	PTPRM	ss1145103439	45903918	FATP, FATWT, HCWT, LMYP, MFY_180D, and MY
25	MTR	ss1145839414	9173663	CVFD_PRI, MFDIAM, MFPER, SL, TESTWT, and UYC
26	DLC1	ss1146742924 ss1146743820 ss1146744870	25957009 26043393	MUSWT, Stature, UDDATT, and WORMCT
Wild sheep				
1	KCNJ10	ss1115230121	117944910	BDENS, BFLUMB3, BONE_WT, FA-C20:5, FA-C22:5, FATP, FECGEN, LMYP, MDLUMB3, MUSWT, and PUFA
1	MGST3	ss1115375784	125162941	BDENS, BFLUMB3, BONE_WT, FA-C20:5, FA-C22:5, FATP, FECGEN, LMYP, MDLUMB3, MUSWT, and PUFA
2	ACO1	ss1121421351 ss1121422525	109766460 109804281	FA-C18:3, FA-C20:4, FA-C20:5, FA-C22:5, HCWT, LATRICH_2, MFPER, and SCS
2	GALNTL6	ss1121533683 ss1121534742 ss1121534985 ss1121536161 ss1121537352 ss1121537767 ss1121538473 ss1121540364 ss1121541584 ss1121544347 ss1121548053 ss1121548153 ss1121548799 ss1121549287 ss1121549759 ss1121554393 ss1121568385 ss1121570596 ss1121571444 ss1121573654 ss1121577773 ss1121580557	116265153 116350674 116362603 116417246 116465257 116500683 116534114 116594826 116635922 116763390 116898149 116905792 116953479 116992107 117072252 117115355 117485350 117573048 117612053 117702396 117736662 117849339	FA-C18:3, FA-C20:4, FA-C20:5, FA-C22:5, HCWT, LATRICH_2, MFPER, and NFEC
2	ZNF395	ss1158590803	111016299	FA-C18:3, FA-C20:4, FA-C20:5, FA-C22:5, HCWT, LATRICH_2, MFPER, and SCS
3	AK8	ss1124538240	4201867	HFEC and SL
3	TSC1	ss1124537252	4132992	HFEC and SL
5	PLIN5	ss1130359135	18089207	FA-C16:1
9	SPAG1	ss1135395144 ss1135395539	84948639 84978362	HCWT, LMA, MFY_180D, and MUSWT
11	NF1	ss1209682579 ss1209684264	44753284 44896884	HCWT, JAWL, and LATRICH_2
12	PRDM16	ss1137575478 ss1137575655	53644681 53663779	FATP and LMYP
13	RALGAPA2	ss1138263148 ss1138263481 ss1138264060 ss1138264441 ss1138264932 ss1138265074 ss1138265616	41284446 41314389 41371630 41394956 41434517 41443275 41483549	MUSWT and SAOS
18	ACAN	ss1218825229	17861995	FA-C20:1, MY, SL, and TESTWT
19	GADL1	ss1219489564	5622653	ASREP and DRESSING

AMDG, age at maximum daily gain; ADG, average daily gain; BFLUMB3, back fat at third lumbar; BW, body weight; BDENS, bone density; BONE_WT, carcass bone weight ; BONEP, carcass bone percentage; FATP, carcass fat percentage; DRESSING, dressing percentage; FATWT, carcass fat weight ; FECGEN, fecal egg count; FCURV, fiber curvature; FLYD, fleece yield; HFEC, *Haemonchus contortus* FEC; HO, horns; HCWT, hot carcass weight; IGA, immunoglobulin A level; IGG, immunoglobulin G level; IOA, inherited ovine arthrogryposis; INTFAT, internal fat amount; JAWL, jaw length; LMYP, lean meat yield percentage; LMA, longissimus muscle area; MFDIAM, mean fiber diameter; FA-C20:4, meat arachidonic acid content; FA-C18:1, meat cis-vaccenic acid content; FA-C22:5, meat docosapentaenoic acid content; FA-C20:5, meat eicosapentaenoic acid content; FA-C20:1, meat gadoleic acid content; FA-C18:2, meat linoleic acid content; FA-C18:3, meat linolenic acid content; FA-C14:0, meat myristic acid content; FA-C18:1, meat oleic acid content; FA-C16:0, meat palmitic acid content; FA-C16:1, meat palmitoleic acid content; PUFA, meat polyunsaturated fatty acid content; FA-C14:0, meat stearic acid content; MCARPL, metacarpal length; MFPER, milk fat percentage; MFY_180D, milk fat yield; MLACT, milk lactose yield; MPUFA, milk polyunsaturated fatty acid content; PP, milk protein percentage; PY, milk protein yield; MYPERS, milk yield persistency; MY, milk yield; MDLUMB3, muscle depth at third lumbar; MUSWT, muscle weight in carcass; NFEC, *Nematodirus* FEC; CVFD_PRI, primary fiber diameter coefficient of variance; RLEGS, rear leg set; ASREP, reproductive seasonality; SAOS, *Salmonella* Abortusovis susceptibility; SCS, somatic cell score; SL, staple length; Stature: stature; SCFA, subcutaneous fat area; SCFT, subcutaneous fat thickness; TESTWT, testes weight; TOTBONE, total bone; LATRICH_2, *Trichostrongylus* adult and larva count; TFEC_1, *Trichostrongylus colubriformis* FEC; UDDATT, udder attachment; UYC, useful yield content; WORMCT, worm count.

## 4 Discussion

The results of the PCA indicated a clear distinction between the wild and domestic sheep populations (Figure 1). The use of genotypic relationship-based PCA has been a valuable tool in various studies to understand population structure and genetic connections between individuals (Sabeti et al., 2007). Domestic samples were collected from different regions, while wild samples were collected from the same region (Taheri et al., 2022). Our analysis revealed a more compact structure within the domestic sheep than the wild sheep, possibly due to intense selection pressure favoring economically desirable traits in domestic sheep (Taheri et al., 2022). However, it is important to note that the limited sample size in this study may lead to an underestimation of the true extent of diversity.

This study used three different methods (iHS, RSB, and XP-EHH) to improve the accuracy of identifying selective sweeps. The wild sheep population showed 135 locations, with the highest iHS values on chromosomes 1, 6, 13, and 18. In contrast, the domestic sheep population had 205 regions, with the strongest signals on chromosomes 2, 3, and 7. Using XP-EHH and FST, 93 candidate genomic regions were identified as harboring putative selective sweeps by Manzari et al. (2019) in three Iranian sheep breeds. The identified signatures of selection were related to multiple candidate genes involved e in skeletal system, energy metabolism, growth, reproduction, and immune and nervous system traits. Eydivandi et al. (2021) applied FST, xp-EHH, Rsb, and FLK tests to identify selective sweeps. Their findings revealed 128, 207, 222, and 252 genomic regions, respectively, as candidates for selective sweeps. Additionally, nine overlapping candidate genes linked to disease resistance and climate adaptation were detected by all four tests. Alipanah et al. (2024) found the highest iHS coefficients under natural selection on chromosomes 3 and 2. Additionally, the XP-EHH results revealed that the highest XP-EHH coefficients under natural selection in European wild sheep, compared to Sardinian wild sheep, were observed on chromosome 3, while the reverse was true for Sardinian wild sheep compared to European wild sheep, with significant findings on chromosome 16.

By analyzing shared regions in the domestic population, we identified the genes *ADGRB3, CADM1, CAPN2, COPG2, DLC1, GALNT10*, *HIP1R, MTR, NEGR1, NRCAM, PML, PTPRM, RELN, RTN1, SDCCAG8,* and *USP25*. These are associated with economic traits such as longissimus muscle area, muscle weight, fecal egg count, milk yield, lean meat yield percentage, carcass fat percentage, bone density, udder attachment, meat palmitoleic acid content, somatic cell score, staple length, useful yield content, carcass bone percentage, meat polyunsaturated fatty acid content, bone weight, and hot carcass weight. *ADGRB3* has been identified as possibly related to fertilization and litter size by regulating oocyte development in Hu sheep (Tao et al., 2021a). In a GWAS study, two significant SNPs within introns of this gene were found to be associated with a reduced number of parasite eggs in feces (Becker et al., 2022). *ADGRB3*, which is linked to the positive regulation of synapse assembly, has been identified in prolific sheep (Hernández-Montiel et al., 2022). *CADM* plays a crucial role in regulating embryo growth, body weight, fat metabolism, and energy balance. It is believed that this gene is involved in determining variations in body size (Xu et al., 2021). *CAPN2* is expressed widely in skeletal muscle, and previous research has linked it with gene expression and meat tenderness in various species (Knight et al., 2012). Ratzka et al. (2008) have shown that the *CAPN2* protein is essential for the normal growth of the preimplantation murine embryo.

*COPG2* was identified in a study of receptor genes related to the progression of Johne’s disease in inbred sheep (Taylor et al., 2008). Additionally, it was found to be more active in the sheep lung and brain on the maternal side prenatally (Duan et al., 2018). Research in cattle revealed that *COPG2* is expressed by both copies during fetal tissue development (Khatib, 2005).

Niciura et al. (2022) identified a link between *GALNT10* and weight traits. Similarly, Al Kalaldeh et al. (2019) found that *GALNT10*, which is responsible for producing a sugar molecule known as mucin-type O-glycan, is associated with the ability of sheep to resist gastrointestinal parasites. Jacobs et al. (2020) revealed that *HIP1R* is crucial for dendrite growth in sheep brain cells. *MTR* contains instructions for producing an enzyme known as 5-methyltetrahydrofolate-homocysteine methyltransferase, which plays a key role in converting homocysteine into methionine—an essential building block for proteins in the body (Zhang et al., 2012). Research suggests that *MTR* influences both the quantity and quality of wool produced by sheep (Rong et al., 2015). *NEGR1* is a cell adhesion molecule that belongs to the LON family of immunoglobulins, which also includes other molecules such as limbic system-associated membrane protein and neurotrimin (Noh et al., 2019). *NEGR1* has been associated with feed efficiency in beef cattle (Seabury et al., 2017) and somatic cell score in sheep (Mohammadi et al., 2022). Specifically, *NEGR1* is more prevalent in the cell adhesion molecular pathway, which plays a role in the body’s defense against disease in cattle (Liu et al., 2020). *NEGR1* is a protein that can be activated as needed and is involved in the growth, specialization, and death of cells in blood vessels (Wang et al., 2021).

*NRCAM* is linked to cells in the nervous system of sheep and with molecules that aid cell adhesion (Alvarez-Franco et al., 2021). Recent studies have demonstrated that *NRCAM*, produced by uterine lining cells, can enhance the body’s response to progestin hormones by altering gene behavior (Cheng et al., 2022). In ruminants, research has revealed that a specific gene called *PML* plays a crucial role in inhibiting tumor growth, indicating a potentially enhanced cancer prevention system in these animals (Wang et al., 2019). Increased expression of *PTPRM* in small intestinal neuroendocrine tumor cells reduces cell growth and division and induces cell death (Barazeghi et al., 2019). *RELN* has been observed to be more expressed in susceptible animals (McRae et al., 2014). Comparing Suffolk and Texel sheep infected with *Teladorsagia circumcincta*, *RELN* was found to be more expressed in Suffolk sheep 3 days post-infection (Ahmed, 2013). Most notably, the expression of *RELN* is concentrated in the theca cells of dominant follicles, where it modulates downstream signaling pathways through paracrine interaction with *LRP8* in granulosa cells (Fayad et al., 2007), regulating the final stages of follicle growth (Nivet et al., 2013). Studies suggest an association between *RELN* and protein kinase activity, contributing to progestogenic pathways, while also highlighting a negative impact of MAP due to *RELN* suppression. *RELN*, a glycoprotein in the extracellular matrix, is involved in various cellular functions, including the MAPK pathway, which is vital for germinal vesicle breakdown and oocyte maturation (Yang et al., 2018). Aboul-Naga et al. (2022) uncovered crucial genetic variations associated with heat tolerance in the gene *RTN1*. *RTN1* is part of the reticulon family residing in the endoplasmic reticulum (ER), which is implicated in hormone release and membrane mobility in nerve cells and is closely linked to ER stress (Chiurchiu et al., 2014). In genome-wide analyses of goats and sheep, *SDCCAG8* was identified as under selection, affecting reproduction and the TGF pathway, which governs the number of offspring in goats and sheep (Tao et al., 2021b). *SDCCAG8* is also involved in cellular component organization (Han M. et al., 2022). Zhang et al. (2022) confirmed that *USP25* is correlated with the annual reproductive cycle in sheep by investigating various sheep breeds with distinct characteristics.

Identifying common segments in the wild population allowed us to accurately locate the genes *ACAN, ACO1, AK8, GADL1, GALNTL6, KCNJ10, MGST3, NF1, PLIN5, PRDM16, RALGAPA2, SPAG1, TSC1,* and *ZNF395*. These genes were associated with several economic traits in the wild population, including muscle weight, milk yield, lean meat percentage, carcass fat percentage, bone density, somatic cell score, staple length, meat polyunsaturated fatty acid content, hot carcass weight, testes weight, milk fat percentage, reproductive seasonality, and jaw length. *ACAN* encodes the aggrecan protein, which is a type of proteoglycan. Aggrecan is the most abundant proteoglycan found in cartilage, a tough and flexible tissue that forms a significant part of the skeleton during early development. The majority of cartilage eventually transforms into bone through a process called ossification, with the exception of the cartilage that remains to cover and protect the ends of bones, along with that found in the nose, airways, and external ears (Mancioppi et al., 2021). *ACAN* mutations can cause skeletal disorders such as osteochondrosis and skeletal dysplasia, which affect height (Sabeti-Aghabozorgi et al., 2022).

Manzari et al. (2019) investigated genes related to skeletal and tail growth, noting that genes such as *ACAN* show signs of selection. *GALNTL6* is an important gene for the ability of sheep to resist gastrointestinal parasites (Pratap et al., 2024). *MGST3* is associated with muscle tissue in sheep and is important for amino acid metabolism (Thameem et al., 2003). *NF1* plays a role in various cellular functions and is linked to sheep reproductive performance. It is crucial for increasing the number of lambs in Texel sheep and for adapting to high-altitude hypoxia (XU et al., 2018). *PLIN5* is involved in fat metabolism and insulin regulation (Zhang et al., 2022). *PRDM16* plays a role in fat cell formation and embryonic development (Chi and Cohen, 2016). *SPAG1* was found to influence fertility in mammals (Faraji et al., 2021). *TSC1* is linked to body size variability in sheep breeds (Cao et al., 2015), while *ZNF395* is associated with growth traits and the development of fat cells. It may also play a role in obesity and metabolic syndrome. *ZNF395* expression levels in Lanzhou fat-tailed sheep were notably higher than in small-tailed Han sheep (Erdenee et al., 2020).

*ADAM9*, *ARHGAP42*, and *CUTC* genes were identified as common genes between domestic and wild sheep species. The *ADAM9* gene plays a crucial role in the nervous, cardiovascular, and muscular systems. In the nervous system, *ADAM9* is involved in neuron migration, axon growth, and synapse formation (Cho, 2012). In the cardiovascular system, it contributes to the proliferation and migration of vascular endothelial cells (Chou et al., 2020). In muscle tissue, *ADAM9* is essential for muscle cell proliferation and differentiation (Ahmed et al., 2017). Furthermore, another study showed significant differences in *ADAM9* expression in sheep skin tissues with varying wool fineness (Tian et al., 2017). The ARHGAP42 gene is associated with hair follicle development and wool production traits in sheep (Sun et al., 2021). This gene regulates angiogenesis in cattle (Diao et al., 2019). Additionally, this gene has been linked to hypertension in a study examining the interaction between genomics and diet in adult males (Imaizumi et al., 2017). The CUTC gene was evaluated in the methane yield metagenome and metatranscriptome datasets of sheep (Kelly et al., 2019).

Quantitative trait locus analysis revealed that significant genes identified in domestic sheep populations were primarily linked to economically important traits, including body weight, bone structure, and muscle development. In contrast, candidate genes found in wild sheep populations were associated with structural and immune traits, such as reproductive behaviors and resistance to parasites, Salmonella abortusovis, and mastitis. Additionally, there were candidate genes in wild sheep related to performance traits, including body weight, muscle growth, milk yield, and milk fat. These results clearly show that selection for higher performance in domestic sheep makes them more sensitive to environmental stressors and diseases, while there is a balance between performance and biologically important traits in wild sheep population. Lv et al. (2021) demonstrated that domestic sheep may have acquired beneficial alleles of various immune and sensory genes through natural or managed hybridization with their wild sheep.

## 5 Conclusion

Our findings suggest that artificial selection by humans, with an emphasis on economically important traits in domestic sheep, has weakened the balance between economic and environmental traits. This balance appears to be crucial for the survival in wild sheep. By identifying potential candidate genes associated with economic and survival traits, along with their genomic regions that have undergone changes due to selection, these insights can be utilized in breeding programs for sheep. However, due to the incomplete information regarding the functional annotation of genes within sheep species and the limited sample size studied, further research with a larger number of samples is necessary to gain a deeper understanding of candidate genes for critical economic traits in both domestic and wild sheep. Advancing knowledge in this area can significantly enhance the design of effective breeding strategies.

## Data Availability

Publicly available datasets were analyzed in this study. These data can be found at: http://projects.ensembl.org/nextgen/.
