# Supplementary material for: Identification of selective sweep and associated QTL traits in Iranian Ovis aries and Ovis orientalis populations
Source: Front Genet. 2024 Dec 19;15:1414717. doi: 10.3389/fgene.2024.1414717 (PMC11693725; doi:10.3389/fgene.2024.1414717)
Supplement: Supplementary file 1 [file Table1.docx]

**TABLE 1 QTLs identified by common regions among iHS, RSB, and XP-EHH methods in domestic sheep**

| CHR | GENE | QTL |
| --- | --- | --- |
| 1 | EAF2 | ASREP, BONE_WT, FA-C20:5, FA-C22:5, FATP, FECGEN, FLYD, LMYP, MDLUMB3, PUFA, SAOS |
| 1 | EIF4G1 | ASREP, BONE_WT, FA-C20:5, FA-C22:5, FATP, FLYD, PUFA, TFEC_1 |
| 1 | FUBP1 | BONE_WT, BONEP, FA-C20:5, FA-C22:5, FATP, LMYP, MUSWT, PUFA |
| 1 | HCLS1 | ASREP, BONE_WT, FA-C20:5, FA-C22:5, FATP, FECGEN, FLYD, LMYP, MDLUMB3, PUFA, SAOS |
| 1 | HUNK | BDENS, BFLUMB3, BONE_WT, FA-C20:5, FA-C22:5, FATP, LMYP, MDLUMB3, MUSWT, PUFA |
| 1 | NMNAT3 | TFEC_1 |
| 1 | OSBPL11 | ASREP, BONE_WT, FA-C20:5, FA-C22:5, FATP, FECGEN, FLYD, LMYP, MDLUMB3, PUFA, SAOS |
| 1 | PCYT1A | ASREP, BONE_WT, FA-C20:5, FA-C22:5, FATP, FECGEN, FLYD, LMYP, MDLUMB3, PUFA, SAOS |
| 1 | SI | ASREP, FA-C22:5, FATP, FCURV, PUFA, TFEC_1 |
| 3 | CAPN13 | SL, TFEC_1 |
| 3 | GALNT14 | SL, TFEC_1 |
| 3 | GXYLT1 | INTFAT, SL |
| 3 | KCNG3 | INTFAT, SL |
| 3 | MGST1 | SL |
| 3 | MTA3 | INTFAT, SL |
| 3 | SPATS2 | INTFAT, PP, SL |
| 3 | XPOT | INTFAT, SL |
| 3 | YAF2 | INTFAT, SL |
| 4 | DPY19L2 | CVFD_PRI |
| 4 | ELMO1 | CVFD_PRI |
| 4 | IMMP2L | CVFD_PRI |
| 4 | SNX10 | CVFD_PRI |
| 4 | TTC26 | CVFD_PRI |
| 5 | ATP10B | FA-C16:1 |
| 5 | KCTD16 | FA-C16:1 |
| 5 | SGCD | FA-C16:1 |
| 6 | GRID2 | FATP, FATWT, FECGEN, HCWT, LMYP, MFDIAM, MUSWT |
| 6 | KCTD8 | FATP, FATWT, FECGEN, LMYP, MFDIAM, MUSWT |
| 6 | SGMS2 | FATP, FATWT, HCWT, LMYP, MFDIAM, MUSWT |
| 6 | TACR3 | FATP, FATWT, HCWT, LMYP, MFDIAM, MUSWT |
| 6 | TNIP3 | FATP, FATWT, INTFAT, LMYP, MFDIAM, MUSWT |
| 7 | AREL1 | CVFD_PRI, SL |
| 7 | BBOF1 | CVFD_PRI, SL |
| 7 | C7H15orf41 | CVFD_PRI, LMA, PP, SL |
| 7 | DCAF4 | CVFD_PRI, SL |
| 7 | DNAAF4 | CVFD_PRI, SL |
| 7 | LIN52 | CVFD_PRI, SL |
| 7 | MEIS2 | CVFD_PRI, LMA, PP, SL |
| 7 | PRTG | CVFD_PRI, SL |
| 7 | RASGRP1 | CVFD_PRI, LMA, PP, SL |
| 7 | RBM25 | CVFD_PRI, SL |
| 7 | TENT2 | CVFD_PRI, LMA, SL |
| 7 | ZFYVE1 | CVFD_PRI, SL |
| 8 | CCDC28A | FECGEN, INTFAT, LATRICH_2 |
| 8 | HECA | FECGEN, INTFAT, LATRICH_2 |
| 8 | NHSL1 | FECGEN, INTFAT, LATRICH_2 |
| 8 | PDE7B | FECGEN, INTFAT, LATRICH_2 |
| 8 | REV3L | INTFAT, LATRICH_2, MFPER |
| 8 | RSPO3 | INTFAT, LATRICH_2 |
| 11 | ARHGAP44 | HCWT, INTFAT, JAWL, LATRICH_2, MPUFA, MY, MYPERS, PY |
| 11 | ARHGEF15 | HCWT, INTFAT, JAWL, LATRICH_2, MPUFA, MY, MYPERS, PY |
| 11 | CHD3 | HCWT, INTFAT, JAWL, LATRICH_2, MPUFA, MY, MYPERS, PY |
| 11 | DNAH2 | HCWT, INTFAT, JAWL, LATRICH_2, MPUFA, MY, MYPERS, PY |
| 11 | PFAS | HCWT, INTFAT, JAWL, LATRICH_2, MPUFA, MY, MYPERS, PY |
| 11 | POLR2A | HCWT, INTFAT, JAWL, LATRICH_2, MPUFA, MY, MYPERS, PY |
| 11 | WRAP53 | HCWT, INTFAT, JAWL, LATRICH_2, MPUFA, MY, MYPERS, PY |
| 12 | CAPN8 | BDENS, FATP, LMYP, MY |
| 12 | RGS7 | FATP, LMYP, MY |
| 13 | GPR158 | MUSWT, SAOS |
| 14 | ADAT1 | BONE_WT, DRESSING, NFEC, TOTBONE |
| 14 | CPNE2 | BONE_WT, DRESSING, FATWT, FECGEN, NFEC, TOTBONE |
| 16 | KCNIP1 | DRESSING, FECGEN, LMYP |
| 16 | RANBP17 | DRESSING, FECGEN, LMYP |
| 17 | DENR | BDENS, SCS |
| 17 | VPS37B | BDENS, SCS |
| 18 | INSM2 | ADG, FA-C20:1, HFEC, SL, TESTWT |
| 18 | LRRK1 | TESTWT |
| 18 | PEAK1 | FA-C20:1, SAOS, SL, TESTWT, WORMCT |
| 18 | RALGAPA1 | ADG, FA-C20:1, HFEC, SL, TESTWT |
| 19 | ARHGEF3 | DRESSING |
| 19 | ERC2 | DRESSING |
| 19 | IL17RD | DRESSING |
| 19 | TASOR | DRESSING |
| 20 | HFE | MY |
| 20 | ICK | IGA, MCARPL, MY, SAOS |
| 20 | JARID2 | MFPER, SCS |
| 20 | KIF6 | MFY_180D, MLACT, MY, PY, SAOS, TESTWT |
| 20 | PLA2G7 | IGA, MCARPL, MFY_180D, MLACT, MY, PY, SAOS, TESTWT |
| 21 | HEPHL1 | FA-C14:0, FA-C16:0, FA-C18:1, FA-C18:2, FA-C18:3, FA-C20:1, FA-C20:4, FA-C22:5, MLACT |
| 21 | TENM4 | ADG, FA-C14:0, FA-C16:0, FA-C18:1, FA-C18:2, FA-C18:3, FA-C20:1, FA-C20:4, FA-C22:5, SAOS |
| 22 | FBXW4 | FECGEN, SCS, TFEC_1 |
| 22 | HECTD2 | SCS |
| 23 | ATP9B | ADG, FATP, FATWT, HCWT, LMYP, MUSWT |
| 23 | MYO5B | MFY_180D, MY |
| 25 | TRIM67 | CVFD_PRI, MFDIAM, MFPER, SL, TESTWT |
| 26 | MYOM2 | MUSWT |
| 26 | TRMT9B | MUSWT, Stature, UDDATT, WORMCT |

**TABLE 2 QTLs identified by common regions among iHS, RSB, and XP-EHH methods in wild sheep**

| CHR | GENE | QTL |
| --- | --- | --- |
| 1 | FOXD2 | ADG, FATP, LMYP, MUSWT |
| 1 | DDR2 | BDENS, BFLUMB3, BONE_WT, FA-C20:5, FA-C22:5, FATP, FECGEN, LMYP, MDLUMB3, MUSWT, PUFA |
| 1 | FAM78B | BDENS, BFLUMB3, BONE_WT, FA-C20:5, FA-C22:5, FATP, FECGEN, LMYP, MDLUMB3, MUSWT, PUFA |
| 1 | FCRL4 | BDENS, BFLUMB3, BONE_WT, FA-C20:5, FA-C22:5, FATP, LMYP, MDLUMB3, MFPER, MUSWT, MY, PP, PUFA |
| 1 | LMX1A | BDENS, BFLUMB3, BONE_WT, FA-C20:5, FA-C22:5, FATP, FECGEN, LMYP, MDLUMB3, MUSWT, PUFA |
| 1 | OLFML2B | BDENS, BFLUMB3, BONE_WT, FA-C20:5, FA-C22:5, FATP, FECGEN, LMYP, MDLUMB3, MUSWT, PUFA |
| 1 | PBX1 | BDENS, BFLUMB3, BONE_WT, FA-C20:5, FA-C22:5, FATP, FECGEN, LMYP, MDLUMB3, MUSWT, PUFA |
| 1 | TIPRL | BDENS, BFLUMB3, BONE_WT, FA-C20:5, FA-C22:5, FATP, LMYP, MDLUMB3, MUSWT, PUFA |
| 1 | UAP1 | BDENS, BFLUMB3, BONE_WT, FA-C20:5, FA-C22:5, FATP, FECGEN, LMYP, MDLUMB3, MUSWT, PUFA |
| 1 | UCK2 | BDENS, BFLUMB3, BONE_WT, FA-C20:5, FA-C22:5, FATP, FECGEN, LMYP, MDLUMB3, MUSWT, PUFA |
| 1 | UHMK1 | BDENS, BFLUMB3, BONE_WT, FA-C20:5, FA-C22:5, FATP, FECGEN, LMYP, MDLUMB3, MUSWT, PUFA |
| 1 | TRABD2B | FATP, LMYP, MUSWT |
| 2 | C2H8orf74 | FA-C18:3, FA-C20:4, FA-C20:5, FA-C22:5, HCWT, LATRICH_2, MFPER, NFEC |
| 2 | C9orf72 | FA-C18:3, FA-C20:4, FA-C20:5, FA-C22:5, HCWT, LATRICH_2, MFPER, SCS |
| 2 | CCDC25 | FA-C18:3, FA-C20:4, FA-C20:5, FA-C22:5, HCWT, LATRICH_2, MFPER, SCS |
| 2 | CLCN3 | FA-C18:3, FA-C20:4, FA-C20:5, FA-C22:5, HCWT, LATRICH_2, MFPER, MLACT, NFEC |
| 2 | DDX58 | FA-C18:3, FA-C20:4, FA-C20:5, FA-C22:5, HCWT, LATRICH_2, MFPER, SCS |
| 2 | ELP3 | FA-C18:3, FA-C20:4, FA-C20:5, FA-C22:5, HCWT, LATRICH_2, MFPER, SCS |
| 2 | MTMR9 | FA-C18:3, FA-C20:4, FA-C20:5, FA-C22:5, HCWT, LATRICH_2, MFPER, NFEC |
| 2 | NEK1 | FA-C18:3, FA-C20:4, FA-C20:5, FA-C22:5, HCWT, LATRICH_2, MFPER, MLACT, NFEC |
| 2 | NUGGC | FA-C18:3, FA-C20:4, FA-C20:5, FA-C22:5, HCWT, LATRICH_2, MFPER, SCS |
| 2 | SCARA3 | FA-C18:3, FA-C20:4, FA-C20:5, FA-C22:5, HCWT, LATRICH_2, MFPER, SCS |
| 2 | SCARA5 | FA-C18:3, FA-C20:4, FA-C20:5, FA-C22:5, HCWT, LATRICH_2, MFPER, SCS |
| 2 | SH3RF1 | FA-C18:3, FA-C20:4, FA-C20:5, FA-C22:5, HCWT, LATRICH_2, MFPER, MLACT, NFEC |
| 3 | ELK3 | ASREP, SL |
| 3 | GLT8D2 | ASREP, NFEC, SL |
| 3 | GNPTAB | ASREP, NFEC, SL |
| 3 | PAH | ASREP, NFEC, SL |
| 3 | FGD6 | BW, IGA, INTFAT, PP, SL |
| 3 | UBE2N | BW, INTFAT, PP, SL |
| 4 | CHCHD3 | CVFD_PRI |
| 5 | HDGFL2 | FA-C16:1 |
| 5 | PTPRS | FA-C16:1 |
| 5 | SH3GL1 | FA-C16:1 |
| 6 | RAP1GDS1 | FATP, FATWT, HCWT, LMYP, MFDIAM, MUSWT |
| 6 | STPG2 | FATP, FATWT, HCWT, LMYP, MFDIAM, MUSWT |
| 6 | TECRL | FATP, FATWT, LMYP, MFDIAM, UYC |
| 7 | AVEN | CVFD_PRI, LMA, SL |
| 7 | DAPK2 | CVFD_PRI, HFEC, LMA, PP, SL |
| 8 | LAMA2 | INTFAT, LATRICH_2 |
| 9 | ANKRD46 | HCWT, LMA, MFY_180D, MUSWT |
| 9 | AZIN1 | HCWT, LMA, MFY_180D, MUSWT |
| 9 | BAALC | HCWT, LMA, MFY_180D, MUSWT |
| 9 | DCAF13 | HCWT, LMA, MFY_180D, MUSWT |
| 9 | NCALD | HCWT, LMA, MFY_180D, MUSWT |
| 9 | RGS22 | HCWT, LMA, MFY_180D, MUSWT |
| 9 | RIMS2 | HCWT, LMA, MFY_180D, MUSWT |
| 9 | RNF19A | HCWT, LMA, MFY_180D, MUSWT |
| 9 | UBR5 | HCWT, LMA, MFY_180D, MUSWT |
| 10 | KLHL1 | BONEP, FATP, FATWT, FECGEN, LMYP, TESTWT |
| 11 | ASIC2 | HCWT, JAWL, LATRICH_2 |
| 11 | BRIP1 | HCWT, LATRICH_2, SAOS |
| 13 | CFAP61 | MUSWT, SAOS |
| 13 | ENTPD6 | MUSWT |
| 13 | KIZ | MUSWT, SAOS |
| 13 | NAPB | MUSWT |
| 13 | RIN2 | MUSWT, SAOS |
| 13 | SLC24A3 | MUSWT, SAOS |
| 13 | TASOR2 | MUSWT |
| 15 | COPB1 | SL |
| 18 | ABHD17C | FA-C20:1, MY, SAOS, SL, TESTWT, WORMCT |
| 22 | PCDH15 | SCS |
| 23 | TSHZ1 | ADG, FATP, FATWT, HCWT, LMYP, MUSWT, RLEGS |
| 23 | ZNF407 | ADG, FATP, FATWT, HCWT, LMYP, MUSWT, RLEGS |
| 23 | SETBP1 | FATP, FATWT, HCWT, LMYP, MFY_180D, MY |
| 24 | TAF6 | BDENS, MFPER, MLACT, MY |

ADG; Average daily gain, BFLUMB3; Backfat at third lumbar, BW; Body weight , BDENS; bone density , BONE_WT; Bone weight in carcass, BONEP; Carcass bone percentage, FATP; Carcass fat percentage, DRESSING; Dressing percentage, FATWT; fat weight in carcass, FECGEN; Faecal egg count, FCURV; Fibre curvature, FLYD; Fleece yield, HFEC; Haemonchus contortus FEC, HCWT; Hot carcass weight, IGA; Immunoglobulin A level, INTFAT; internal fat amount, JAWL; Jaw length, LMYP; Lean meat yield percentage, LMA; Longissimus muscle area, MFDIAM; Mean fibre diameter, FA-C20:4; Meat arachidonic acid content, FA-C18:1; Meat cis-vaccenic acid content, FA-C22:5; Meat docosapentaenoic acid content, FA-C20:5; Meat eicosapentaenoic acid content, FA-C20:1; Meat gadoleic acid content, FA-C18:2; Meat linoleic acid content, FA-C18:3; Meat linolenic acid content, FA-C14:0; Meat myristic acid content, FA-C18:1; Meat oleic acid content, FA-C16:0; Meat palmitic acid content, FA-C16:1; Meat palmitoleic acid content, PUFA; Meat polyunsaturated fatty acid content, FA-C14:0; Meat stearic acid content, MCARPL; Metacarpal length, MFPER; Milk fat percentage, MFY_180D; Milk fat yield, MLACT; milk lactose yield, MPUFA; milk polyunsaturated fatty acid content, PP; Milk protein percentage, PY; Milk protein yield, MYPERS; Milk yield persistency, MY; Milk Yield, MDLUMB3; Muscle depth at third lumbar, MUSWT; muscle weight in carcass, NFEC; Nematodirus FEC, CVFD_PRI; Primary fibre diameter coefficient of variance, RLEGS; Rear leg set, ASREP; Reproductive seasonality, SAOS; Salmonella abortusovis susceptibility, SCS; Somatic Cell Score, SL; Staple length, Stature; Stature, TESTWT; Testes weight, TOTBONE; Total bone, LATRICH_2; Trichostrongylus adult and larva count, TFEC_1; Trichostrongylus colubriformis FEC, UDDATT; udder attachment, UYC; Useful yield content, WORMCT; Worm count
